# Deubiquitylase OTUD1 confers Erlotinib sensitivity in non-small cell lung cancer through inhibition of nuclear translocation of YAP1

**DOI:** 10.1038/s41420-022-01119-w

**Published:** 2022-10-01

**Authors:** Huafeng Liu, Liting Zhong, Yanjun Lu, Xuewen Liu, Jiawang Wei, Yuhai Ding, Huiling Huang, Qihong Nie, Xiaohong Liao

**Affiliations:** Department of Oncology, Ganzhou People’s Hospital, Ganzhou, 341000 P. R. China

**Keywords:** Non-small-cell lung cancer, Non-small-cell lung cancer

## Abstract

Evidence exists suggesting tumor-inhibiting properties of deubiquitylase OTUD1 in various malignancies. We herein investigated the anti-tumor effect and clarified the downstream mechanisms of OTUD1 in the chemoresistance of non-small cell lung cancer (NSCLC) cells. Expression of OTUD1 was examined in NSCLC (PC-9 cells) and erlotinib-resistant NSCLC (PC-9/ER) cell lines. OTUD1 was bioinformatically predicted to be weakly expressed in NSCLC tissue samples and verified in PC-9/ER cells. PC-9/ER cells were subsequently subjected to ectopic expression of OTUD1 alone or combined with SOX9 to dissect out the effect of OTUD1 on the proliferation, chemoresistance and apoptosis in vitro and in vivo. OTUD1 upregulation sensitized NSCLC cells to erlotinib both in vitro and in vivo. In the presence of OTUD1 overexpression, nuclear translocation of YAP1 was inhibited and its expression was inactivated. This effect of OTUD1 was associated with the decreased ubiquitination level of YAP1. SOX9/SPP1 inactivation was the consequence of inhibited nuclear translocation of YAP1. Overexpression of SOX9 reversed the inhibitory effect of OTUD1 on the resistance of NSCLC cells to erlotinib. In conclusion, our study reveals that OTUD1 potentially acts as a tumor suppressor and suppresses erlotinib resistance of NSCLC through the YAP1/SOX9/SPP1 axis, suggesting that OTUD1 may serve as a target for reducing chemoresistance for NSCLC.

## Introduction

Lung cancer is the most frequently occurring cause of cancer-related mortality across the globe [1]. Non-small cell lung cancer (NSCLC) is a most common form of lung cancer accounting for 85% of lung cancers [2]. Being a heterogeneous disease at molecular levels, comprehensive insights for its biologic characteristics is pivotal to discover effective treatment options [3]. Erlotinib, a EGFR tyrosine kinase inhibitor (TKI), enables improved survival in NSCLC patients, but the treatment outcome is limited by drug resistance [4]. A better understanding of the molecular mechanistic basis behind chemoresistance enables development of targeted therapies of personalized medicine.

OTUD1 is a deubiquitylase belonging to the OTU family and has been detected in multiple types of human cancers [5, 6]. Recent evidence has highlighted the host anti-tumor immunity of OTUD1 as it can potentiate the release of damage-associated molecular patterns, which in turn recruits the leukocytes and strengthens host immune response [7]. High mRNA expression of OTUD1 indicates the improved prognosis in NSCLC [8]. Additionally, OTUD1 can represent a target to overcome the chemoresistance in pancreatic ductal adenocarcinoma cells [9]. Knockdown of OTUD1 results in nuclear localization of yes-associated protein (YAP) and the resultant activation of its expression [10]. It is interesting to note that YAP1 is a critical downstream effector along the Hippo signaling implicated in carcinogenesis and malignant phenotypes, as well as resistance to anti-tumor therapies [11]. Expression of YAP1 is elevated in NSCLC samples and this elevation can facilitate the oncogenic phenotypes of NSCLC [12]. Meanwhile, YAP activation can induce tetraploidization and thus enhance gefitinib resistance in NSCLC [13].

Direct interaction with and activation of YAP1 is capable pf inducing the transcription of SOX9 [14]. Recent evidence suggests that SOX9 is associated with clinical tumor-node-metastasis stage and it can stimulate the malignant biological characteristics of NSCLC in vitro [15]. Meanwhile, upregulated SOX9 can enhance gefitinib resistance of lung cancer, corresponding to increased cancer cell oncogenic phenotypes and gefitinib intake [16]. SOX9 is a transcriptional mediator of SPP1 in hepatocytes where SOX9 can activate the expression of SPP1 [17]. SPP1, also known as osteopontin, has the potential to predict the response of NSCLC cells to first-line platinum-based chemotherapy [18]. Herein, we made efforts in examining the hypothesized regulatory effects of OTUD1 on the NSCLC cell growth dynamics and chemoresistance, and clarifying the potential anti-cancer network of OTUD1/YAP1/SOX9/SPP1.

## Results

### OTUD1 expression is downregulated in NSCLC samples

**Fig. 1:**
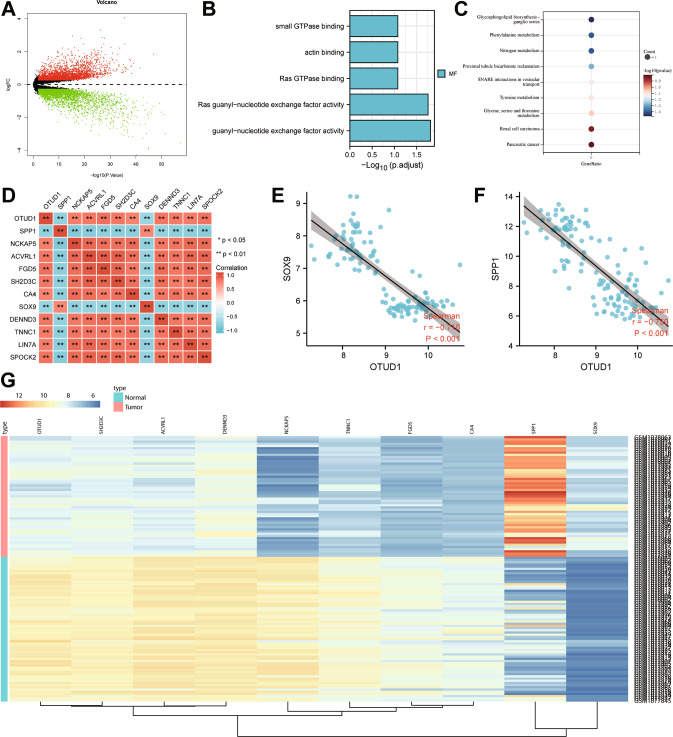
Prediction of the DEGs and their molecular interactions in NSCLC. **A** A volcano map of the results from differential analysis of the GSE44077 dataset. Red dots represent upregulated genes, green dots represent downregulated genes and black dots represent un-differentially expressed genes. **B** GO analysis of the top 50 DEGs screened from the GSE44077 dataset. **C** KEGG enrichment analysis of the top 50 DEGs screened from the GSE44077 dataset. **D** Correlation analysis heat map of the top 10 DEGs screened from the GSE44077 dataset. **E** Correlation analysis of OTUD1 and SOX9 in the GSE44077 dataset. **F** Correlation analysis of OTUD1 and SPP1 in the GSE44077 dataset. **G** A heat map of the expression of the top 10 differential genes in the GSE44077 dataset. The color scale from green to red indicates the expression level of genes from low to high.

Differential analysis of the GSE44077 dataset revealed 3573 significantly upregulated and 3266 downregulated mRNAs in the NSCLC tissue samples (Fig. 1A). The top 50 differentially expressed genes (DEGs) were selected for GO and KEGG enrichment analysis, which depicted that these genes were mainly related to the occurrence of cancer (Fig. 1B, C).

In addition, correlation analysis heat map of the top 10 DEGs indicated that OTUD1 was negatively correlated with SOX9 and SPP1 (Fig. 1D). Further correlation analysis demonstrated an adverse correlation between OTUD1 and SOX9 (r = −0.71, *p* < 0.001) (Fig. 1E), as well as between OTUD1 and SOX9 (r = −0.71, *p* < 0.001) (Fig. 1F). A heat map of the top 10 DEGs is summarized in Fig. 1G where the expression of OTUD1 was significantly lower while that of SOX9 and SPP1 was higher in NSCLC tissue samples than that in normal tissue samples.

The above bioinformatics analysis results indicated that OTUD1 was poorly expressed in NSCLC samples and may participate in the occurrence of NSCLC through SOX9 and SPP1.

### OTUD1 suppresses resistance of NSCLC cells to erlotinib

**Fig. 2:**
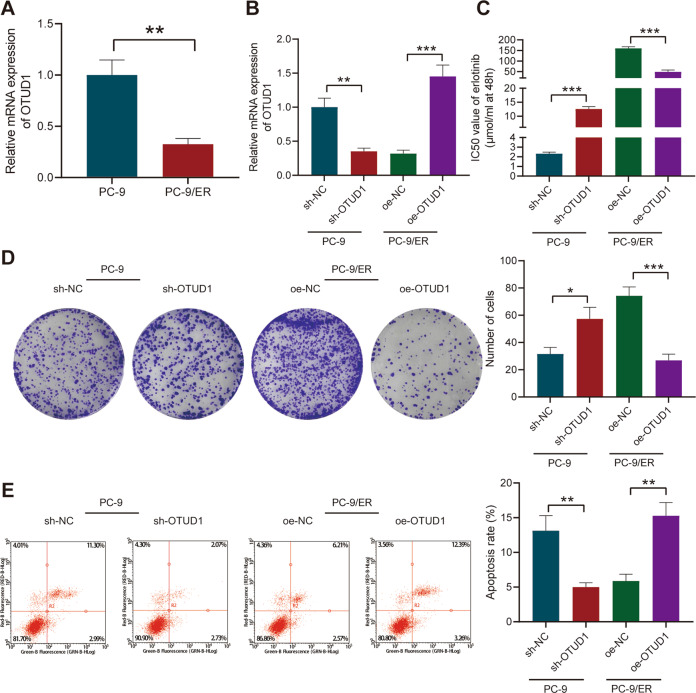
OTUD1 attenuates the resistance of NSCLC cells to erlotinib. **A** OTUD1 expression in PC-9/ER and PC-9 cells determined by RT-qPCR. **B** Transduction efficiency of oe-OTUD1 in PC-9/ER cells and sh-OTUD1 in PC-9 cells determined by RT-qPCR. **C** IC50 value of erlotinib in oe-OTUD1-treated PC-9/ER cells and sh-OTUD1-treated PC-9 cells determined by CCK-8 assay. **D** Clonogenic potential of oe-OTUD1-treated PC-9/ER cells and sh-OTUD1-treated PC-9 cells determined by colony formation assay. **E** Apoptosis rate of oe-OTUD1-treated PC-9/ER cells and sh-OTUD1-treated PC-9 cells measured by flow cytometry. *p < 0.05, **p < 0.01, ***p < 0.001. Data are shown as mean ± standard deviation of three technical replicates. Data between two groups were compared by unpaired *t* test.

Effect of OTUD1 on the resistance of NSCLC cells to erlotinib was then analyzed. Lower expression of OTUD1 was found in PC-9/ER cells versus that in PC-9 cells (Fig. 2A). Thus, OTUD1 was overexpressed in PC-9/ER cells using lentivirus overexpressing OTUD1 (oe-OTUD1) while silenced in PC-9 cells using short hairpin RNA (shRNA) against OTUD1 (sh-OTUD1) (Fig. 2B).

Cell counting kit-8 (CCK-8) assay showed a reduction in the half maximal inhibitory concentration (IC50) value of erlotinib upon OTUD1 overexpression in PC-9/ER cells while OTUD1 silencing led to opposite results in PC-9 cells (Fig. 2C). Furthermore, the results of colony formation assay and flow cytometry explained that overexpression of OTUD1 in PC-9/ER cells inhibited cell proliferation whereas augmenting cell apoptosis, but knockdown of OTUD1 in PC-9 cells caused opposite results (Fig. 2D, E).

Collectively, these data demonstrated that overexpression of OTUD1 could reverse erlotinib resistance in NSCLC cells.

### OTUD1 suppresses resistance to erlotinib in NSCLC tumor-bearing mice

**Fig. 3:**
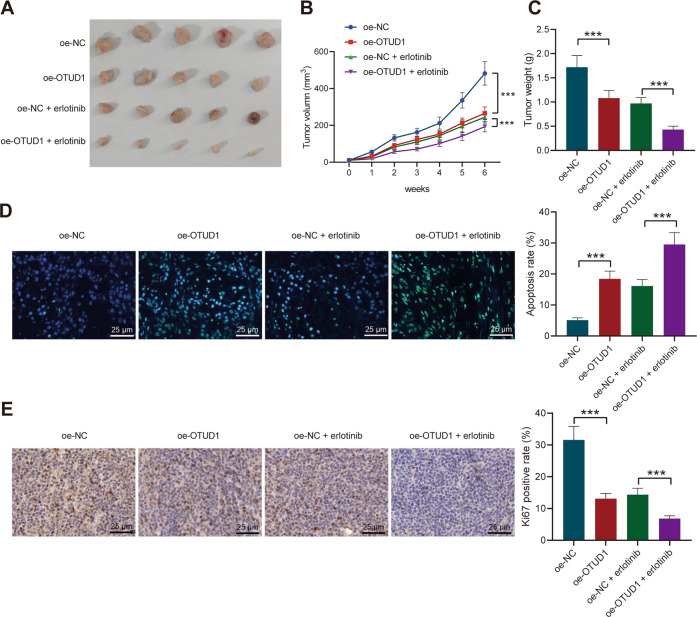
OTUD1 attenuates the resistance of NSCLC cells to erlotinib in vivo. Mice were inoculated with PC-9/ER cells transduced with lentivirus carrying oe-OTUD1, or treated with oe-OTUD1 + erlotinib. **A** Representative images showing xenografts in mice. **B** Tumor volume of mice. **C** Tumor weight of mice. **D** TUNEL positive cells in the tumor tissues of mice. **E** IHC staining of Ki67 protein in the tumor tissues of mice. *n* = 10. ***p < 0.001. Data are shown as mean ± standard deviation. Data among multiple groups were analyzed by one-way ANOVA and Tukey’s multiple comparisons test. Bonferroni-corrected repeated measures ANOVA was applied for comparison of data at varied time points.

We then moved to further determining the effect of OTUD1 on the resistance of NSCLC cells to erlotinib in vivo. The mice were inoculated with PC-9/ER cells transduced with lentivirus. The tumor volume and weight of the oe-OTUD1-treated mice were significantly reduced with or without erlotinib treatment (Fig. 3A–C).

TUNEL assay results exhibited an increase in cell apoptosis upon OTUD1 overexpression. In presence of erlotinib, OTUD1 overexpression further promoted cell apoptosis (Fig. 3D). Immunohistochemical (IHC) results illustrated that Ki67 positive cells were fewer in the tumor tissues of mice treated with oe-OTUD1 while much more fewer Ki67 positive cells were detected when OTUD1 was overexpressed in the presence of erlotinib (Fig. 3E).

In summary, overexpression of OTUD1 can inhibit tumorigenicity of and reverse resistance of NSCLC cells to erlotinib in vivo.

### OTUD1 inhibits nuclear translocation of YAP1 through deubiquitination

**Fig. 4:**
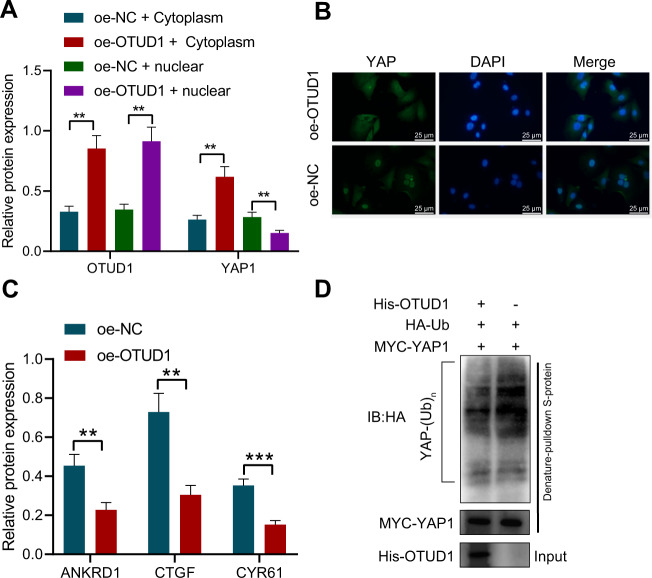
OTUD1 arrests nuclear translocation of YAP1 through deubiquitination and reduces the activity of YAP1. **A** Western blot analysis of YAP1 protein in the nucleus and cytoplasm of PC-9/ER cells overexpressing OTUD1. **B** Immunofluorescence detection of YAP1 localization in the PC-9/ER cells overexpressing OTUD1. **C** Western blot analysis of ANKRD1, CTGF and CYR61 proteins in PC-9/ER cells overexpressing OTUD1. **D** Ubiquitination level of YAP1 in PC-9/ER cells overexpressing OTUD1 detected by Co-IP assay. **p < 0.01, ***p < 0.001. Data are shown as mean ± standard deviation of three technical replicates. Data between two groups were compared by unpaired *t* test.

Next, we attempted to elucidate the mechanism underlying the inhibitory effect of OTUD1 on the resistance of NSCLC cells to erlotinib. As shown in Fig. 4A, oe-OTUD1 in PC-9/ER cells decreased YAP1 expression in the nucleus while increasing YAP1 expression in the cytoplasm. The results of immunofluorescence detection showed that YAP1 was transferred from the nucleus to the cytoplasm upon overexpression of OTUD1 (Fig. 4B). In addition, overexpression of OTUD1 downregulated the expression of the classic YAP1 target genes ANKRD1, CTGF and CYR61 (Fig. 4C), indicating that OTUD1 overexpression can inhibit the activity of YAP1.

Meanwhile, the ubiquitination level of YAP1 was reduced following overexpression of OTUD1 as detected by Co-immunopreciptation (Co-IP) assay in PC-9/ER cells (Fig. 4D).

The above results indicated that OTUD1 may inhibit nuclear translocation of YAP1 through deubiquitination, thereby inhibiting the activity of YAP1.

### OTUD1 reverses erlotinib resistance in NSCLC cells by inactivating the SOX9/SPP1 axis

**Fig. 5:**
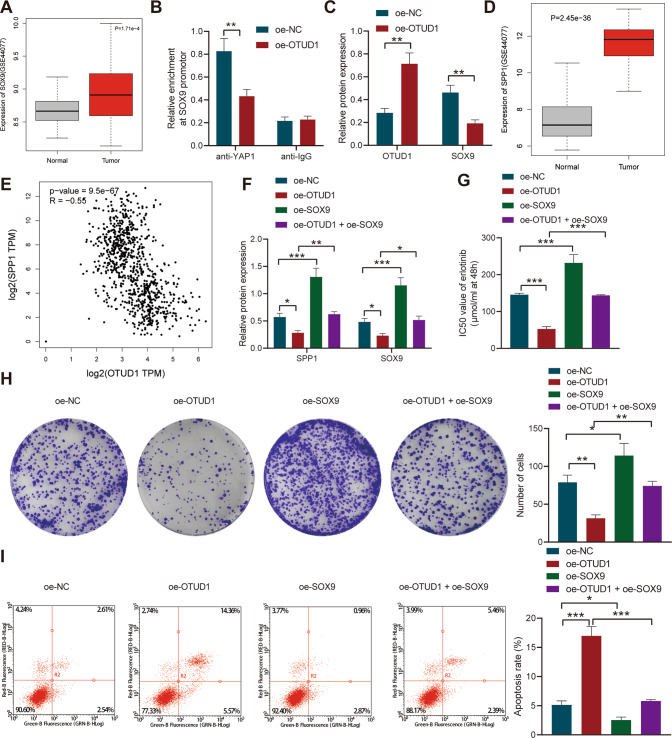
OTUD1 suppresses resistance of NSCLC cells to erlotinib by inactivating the SOX9/SPP1 axis. **A** Box plots of SOX9 expression in normal and NSCLC tissue samples in the GSE44077 dataset. **B** The enrichment of YAP1 in the SOX9 promoter region analyzed by ChIP assay. **C** Western blot analysis of SOX9 protein in the oe-SOX9-treated PC-9/ER cells. **D** Box plots of SPP1 expression in normal and NSCLC tissue samples in the GSE44077 dataset. **E** Correlation analysis of OTUD1 with SPP1 in TCGA database. **F** Western blot analysis of SPP1 and SOX9 proteins in PC-9/ER cells treated with oe-OTUD1, oe-SOX9 or both. **G** IC50 value of erlotinib in PC-9/ER cells treated with oe-OTUD1, oe-SOX9 or both determined by CCK-8 assay. **H** Clonogenic potential of PC-9/ER cells treated with oe-OTUD1, oe-SOX9 or both determined by colony formation assay. **I** Apoptosis rate of PC-9/ER cells treated with oe-OTUD1, oe-SOX9 or both measured by flow cytometry. *p < 0.05, **p < 0.01, ***p < 0.001. Data are shown as mean ± standard deviation of three technical replicates. Data among multiple groups were compared by one-way ANOVA and Tukey’s multiple comparisons test.

Differential analysis of the GSE44077 dataset showed that SOX9 was overexpressed in NSCLC tissue samples (Fig. 5A). Chromatin immunoprecipitation (ChIP) experiment results revealed that overexpression of OTUD1 inhibited the enrichment of YAP1 in the SOX9 promoter region (Fig. 5B). Therefore, it was concluded that OTUD1 may inhibit the interaction between YAP1 and SOX9 by delaying the nuclear translocation of YAP1.

Western blot analysis results showed a decline in the expression of SOX9 in the presence of OTUD1 overexpression in PC-9/ER cells (Fig. 5C). In addition, box plots on analysis results of the GSE44077 dataset suggested significantly high expression of SPP1 in the NSCLC tissue samples (Fig. 5D). Meanwhile, retrieval of TCGA database found that OTUD1 and SPP1 were inversely correlated (Fig. 5E). Hence, SOX9 was further overexpressed in PC-9/ER cells. Western blot analysis results confirmed that the expression of SOX9 and SPP1 was reduced in the oe-OTUD1-treated cells while it was elevated upon oe-SOX9 treatment. Dual treatment with oe-OTUD1 and oe-SOX9 elevated the expression of SOX9 and SPP1 when compared with oe-OTUD1 treatment alone (Fig. 5F).

The results of CCK-8 assay (Fig. 5G), colony formation assay (Fig. 5H) and flow cytometry (Fig. 5I) exhibited a reduction in the IC50 value of erlotinib and cell proliferation and an increase in cell apoptosis following oe-OTUD1 treatment, while the effect induced by oe-SOX9 treatment turned out to be opposite. Also, oe-SOX9 treatment reversed the results triggered by oe-OTUD1 treatment.

These lines of evidence demonstrated that OTUD1 can impair the resistance of NSCLC cells to erlotinib by inhibiting the SOX9/SPP1 axis, which was rescued by SOX9 overexpression.

## Discussion

Our work provided new mechanistic insights for the inhibitory property of OTUD1 on the chemoresistance of NSCLC to erlotinib *via* suppression of YAP1 nuclear translocation and inactivation of the SOX9/SPP1 axis.

The evidence provided by our study highlighted that OTUD1 under-expression occurred in PC-9/ER cells, while its ectopic expression attenuated the resistance of NSCLC cells to erlotinib based on in vitro and in vivo experiments. Consistently, lowered expression of OTUD1 has also been documented in lung cancer samples [8]. Recent data have demonstrated the central role of OTUD1 in activating caspase-independent and caspase-dependent apoptosis and that decreased OTUD1 expression contributes to promotion of chemoresistance in esophageal squamous cell carcinoma [19]. Subsequent results of the current study revealed that OTUD1 may inhibit nuclear translocation of YAP1 through deubiquitination, thereby inhibiting the activity of YAP1. In line with this, forced expression of OTUD1 has been shown to retain cytoplasmic YAP expression and consequently represses its activity, meanwhile, ablation of OTUD1 results in YAP nuclear translocation and activation [10]. Evidence has been presented documenting that YAP1 suppression causes a decline in oncogenic characteristics of tumors and an elevation in the therapeutic sensitivity, which points to the potential for targeting YAP1 as a novel therapeutic approach in malignancies [11]. YAP1 activation induces multidrug resistance by inhibiting the apoptosis of SCLC while its deficiency increases drug sensitivity in vitro and in vivo [20]. Corroborating findings are identified in a previous study, which demonstrated that the aberrant nuclear accumulation of YAP1 leads to carcinogenesis [21]. Thus, it is reasonable to suggest based on the aforementioned findings that OTUD1 impaired NSCLC cell growth and erlotinib resistance by suppressing the nuclear translocation of YAP1.

Further mechanistic findings suggested that OTUD1 may inhibit the SOX9/SPP1 axis by delaying the nuclear translocation of YAP1, thus reversing the resistance of NSCLC cells to erlotinib. The positive correlation between the YAP signaling and SOX9 expression has been observed in esophageal squamous cell carcinoma, where YAP transcriptionally activates its downstream target SOX9 [22]. Upregulation of SOX9 can impair the inhibition of NSCLC cell proliferative and invasive capacities as well as resistance to apoptosis caused by forced expression of microRNA-133b [23]. In addition, a recent study has highlighted overexpression of SOX9 expression in NSCLC cells treated with chemotherapeutic cisplatin, while SOX9 knockdown results in augmented cisplatin sensitivity of NSCLC [24]. SPP1 is a downstream target of SOX9 which results in elevated serum SPP1 levels and SPP1 can serve as a useful surrogate marker of SOX9 in hepatocellular carcinoma [25]. SSP1 is expressed at high levels in lung cancer tissues as well as in afatinib-resistant lung cancer cells whereas its knockdown sensitizes lung cancer cells to afatinib and decreases cell invasion [26]. These findings indicate that OTUD1-mediated YAP1/SOX9/SPP1 axis may deem as a potential option for the treatment of NSCLC.

**Fig. 6:**
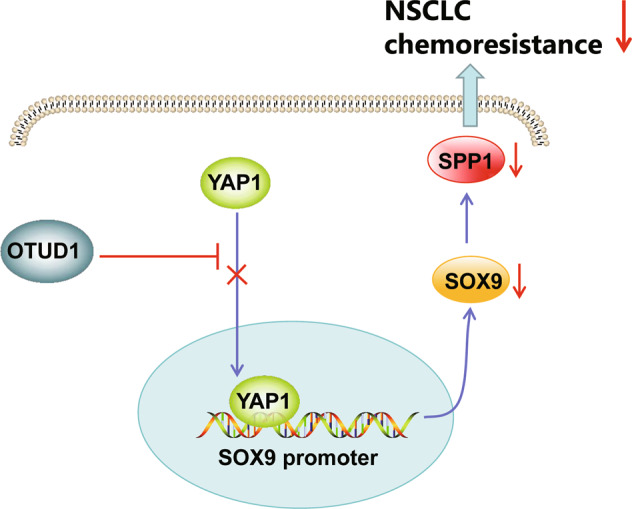
Schematic diagram of the mechanism by which OTUD1 affects the resistance of NSCLC cells to erlotinib. OTUD1 inhibits the nuclear translocation of YAP1 and inactivates the SOX9/SPP1 axis, ultimately reversing the resistance of NSCLC cells to erlotinib.

To summarize all that we have gathered, OTUD1 reversed NSCLC cell resistance to erlotinib via inactivation of the SOX9/SPP1 axis by inhibiting the nuclear translocation of YAP1, underlying a novel mechanism of the anti-oncogenic OTUD1 in NSCLC (Fig. 6). However, the potential role of proteins belonging to deubiquitinase subfamily in the prognostic evaluation of gefitinib sensitivity in NSCLC models could be further explored. Moreover, additional studies focusing on the functional relevance of OTUD1 in resistance of NSCLC cells to the first or second generation TKIs are warranted in the future.

## Materials and methods

### Microarray-based assay

NSCLC-related microarray data GSE44077 [27] (66 adjacent tissue samples and 55 NSCLC tissue samples) was retrieved from GEO database. With |logFC | > 1.5, and *p* < 0.01 as the threshold, R “limma” package was adopted for differential analysis to identify DEGs in NSCLC tissue samples.

### Cell culture

Human NSCLC cell line PC-9 purchased from (Biobw, Beijing, China) was cultured in RPMI-1640 medium (Gibco) containing 10% FBS, 100 U/mL penicillin and 10 μg/mL streptomycin in a 5% CO_2_ incubator at 37 °C.

To develop erlotinib-resistant cell line PC-9/ER, parental PC-9 cells were cultured in the medium supplemented with erlotinib (S1023, Selleck), concentration of which was gradually increased until 10 nmol/L as the final concentration. Then, cells were cultured in fresh drug-free RPMI-1640 for 24 h. When the living cells reached 80% confluence, erlotinib was added until concentration reached 1 μmol/L [28, 29].

### Lentiviral transduction

PC-9 and PC-9/ER cells were then transduced with lentivirus (Genechem, Shanghai, China) carrying oe-NC, oe-OTUD1, sh-NC, sh-OTUD1 or oe-SOX9. The cells were cultured with 10 mL of virus medium mixture and 5 μL Polybrene overnight with 5% CO_2_ at 37 °C. Upon the cells settled at the bottom of the dish, a medium containing 2 μg/mL puromycin was supplemented for screening.

### Gene expression quantitation

Total RNA was extracted from cells with TRIzol reagent (15596026, Invitrogen). RNA was then reversely transcribed into cDNA by PrimeScript RT reagent Kit (RR047A, Takara, Japan). With SYBR Green fluorescence dye (RR091A, Takara), RT-qPCR was followed on an ABI 7500 qPCR instrument. As normalized to GAPDH, fold changes were quantified using 2^−ΔΔCt^ (Supplementary Table 1).

### Protein expression quantitation

Enhanced RIPA lysis buffer containing protease inhibitor was adopted to extract total protein, followed by concentration quantification by a BCA kit (Boster). Extracts were resolved and electro-transferred onto membranes. After blocking in 5% BSA, membranes were probed overnight at 4 °C with primary rabbit antibodies against OTUD1 (1: 1000, PA5-107207, Invitrogen), SOX9 (1: 1000, ab185966, Abcam, Cambridge, UK), Ki67 (1: 1000, A11390, ABclonal), ANKRD1 (1: 1000, A6192, ABclonal), CTGF (1: 1000, A11067, ABclonal), CYR61 (1: 1000, A1111, ABclonal), SPP1 (1: 1000, 214050, ABclonal), GAPDH (1: 1000, ab8227, Abcam; internal control), and YAP1 (1: 1000, 52771, Abcam) and then with HRP-labeled secondary antibody (goat anti-rabbit IgG, ab205719, 1: 2000, Abcam) for 1 h. ECL reagent (EMD Millipore, Billerica, MA) was adopted to visualize the proteins, with band intensities quantified by Image J software.

### CCK-8 assay

IC50 values of cells to erlotinib were determined using CCK-8 kit (Dojindo Laboratories, Kumamoto, Japan). Cells were treated for 48 h with PBS-diluted erlotinib at different concentrations (0, 0.5, 1, 2, 4, 6 μmol/L in detection of PC-9 cells; 0, 100, 120, 150, 180, 200 μmol/L in detection of PC-9/ER cells). Each well underwent incubation with 110 μL CCK-8 solution (100 μL RPMI-1640, 10 μL CCK-8 reagent). The OD value quantification was conducted at 450 nm by a microplate reader.

### Cellular immunofluorescence

After 4% paraformaldehyde fixation and 0.5% Triton X-100 incubation. Cells were immunostained overnight with anti-YAP1 antibody (1: 100, ab52771, Abcam). The second day, the cells were incubated with fluorescent secondary antibody (1: 1000) and visualized under a fluorescence microscope.

### Flow cytometry and colony formation assay

Flow cytometry using a apoptosis detection kit (BD Pharmingen, San Diego, CA) was adopted to assess cell apoptosis. Cells underwent incubation with 5 μL Annexin V-FITC and 5 μL PI on a flow cytometer. In terms of colony formation assay, cell suspension was plated in a 6-well plate (500 cells/well) and cultured in a 5% CO_2_ incubator at 37 °C. Upon the cells adhering to the wall, the colony formation was observed every day for 7–10 days. After crystal violet staining, colonies were counted with a microscope.

### Co-IP assay

PC-9/ER cells were transfected for 48 h with HA-Ubiquitination and MYC-YAP1 plasmids, respectively. S-protein beads pulled MYC-YAP1 down, whereupon the cells underwent incubation with bacterially purified His-OTUD1 protein in deubiquitination buffer for 3 h. Beads were washed with deubiquitination buffer, and the bound protein was eluted, followed by Western blot with tag antibodies against MYC (1: 1000, #2276, CST, Beverly, MA) and His (1: 1000, #12698, CST).

### ChIP

The EZ-Magna ChIP kit (EMD Millipore) was adopted for ChIP assay. DNA-protein cross-linking was produced by formaldehyde incubation. The cells were then lysed and subjected to ultrasonication to produce 200–300 bp chromatin fragments. The antibodies used in the experiment included YAP1 (1: 50, #14074, CST) and NC IgG (#3900, CST). Finally, enrichment of YAP1 in SOX9 promoter region was assessed by qPCR.

### Xenograft tumor in nude mice

Forty 6-to-8-week healthy BALB/c nude mice (Beijing Vital River Laboratory Animal Technology Co., Ltd., Beijing, China) were housed individually in the specific pathogen free laboratory (60–65% humidity, 22–25 °C, and a 12-h light/dark cycle), with free access to water and food for one-week acclimatization before experiment. The current study was performed with the approval of the Animal Ethics Committee of Ganzhou People’s Hospital, in accordance with the Guide for the Care and Use of Laboratory animals published by the US National Institutes of Health.

The mice were inoculated with PC-9/ER cells transduced with lentivirus harboring oe-NC and oe-OTUD1, or treated with oe-NC + erlotinib and oe-OTUD1 + erlotinib, with 10 mice upon each treatment. Upon tumor volume reaching 10 mm^3^, mice received intraperitoneal injection of 50 mg/kg erlotinib twice every week for 6 weeks. At the end of the experiment, mice were euthanized and the tumor was removed with the size and weight measured.

### IHC staining

After antigen retrieval and normal goat serum blocking, tumor tissue sections were probed overnight at 4 °C with primary antibody against Ki67 (mouse anti-human, 1: 200, ab15580, Abcam), followed by incubation at 37 °C for 20 min with secondary antibody against goat anti-rabbit IgG (1: 1000, #7076, CST). Following treatment with HRP-labeled streptavidin protein working solution and DAB (ST033, Whiga, Guangzhou, China) development, sections were photographed with a microscope.

### TUNEL assay

The apoptosis of NSCLC cells in mouse tumor tissues was measured according to one-step TUNEL cell apoptosis detection kit (C1086, Beyotime, Shanghai, China). The tumor tissue sections were incubated with 50 μL TUNEL solution and the nucleus was stained with DAPI. The tissue sections were subjected to inverted microscopic observation of TUNEL-positive cells.

### Statistical analysis

Statistical analyses included unpaired *t*-test was adopted to compare two-group data, one-way analysis of variance (ANOVA) with Tukey’s post-hoc test to compare multi-group data and bonferroni-corrected repeated measures ANOVA to compare variables at varied time points. All results were processed using SPSS 22.0 (IBM Corp., Armonk, NY), presented as mean ± standard deviation. *p* < 0.05 was statistically significant.

## Supplementary information

Original Data File

Supplementary Table 1

## Data Availability

The datasets generated and/or analysed during the current study are available in the manuscript and supplementary materials.
