# Supplementary figures and images for: Deubiquitylase OTUD1 confers Erlotinib sensitivity in non-small cell lung cancer through inhibition of nuclear translocation of YAP1
Source: Cell Death Discov. 2022 Oct 1;8:403. doi: 10.1038/s41420-022-01119-w (PMC9526728; doi:10.1038/s41420-022-01119-w)

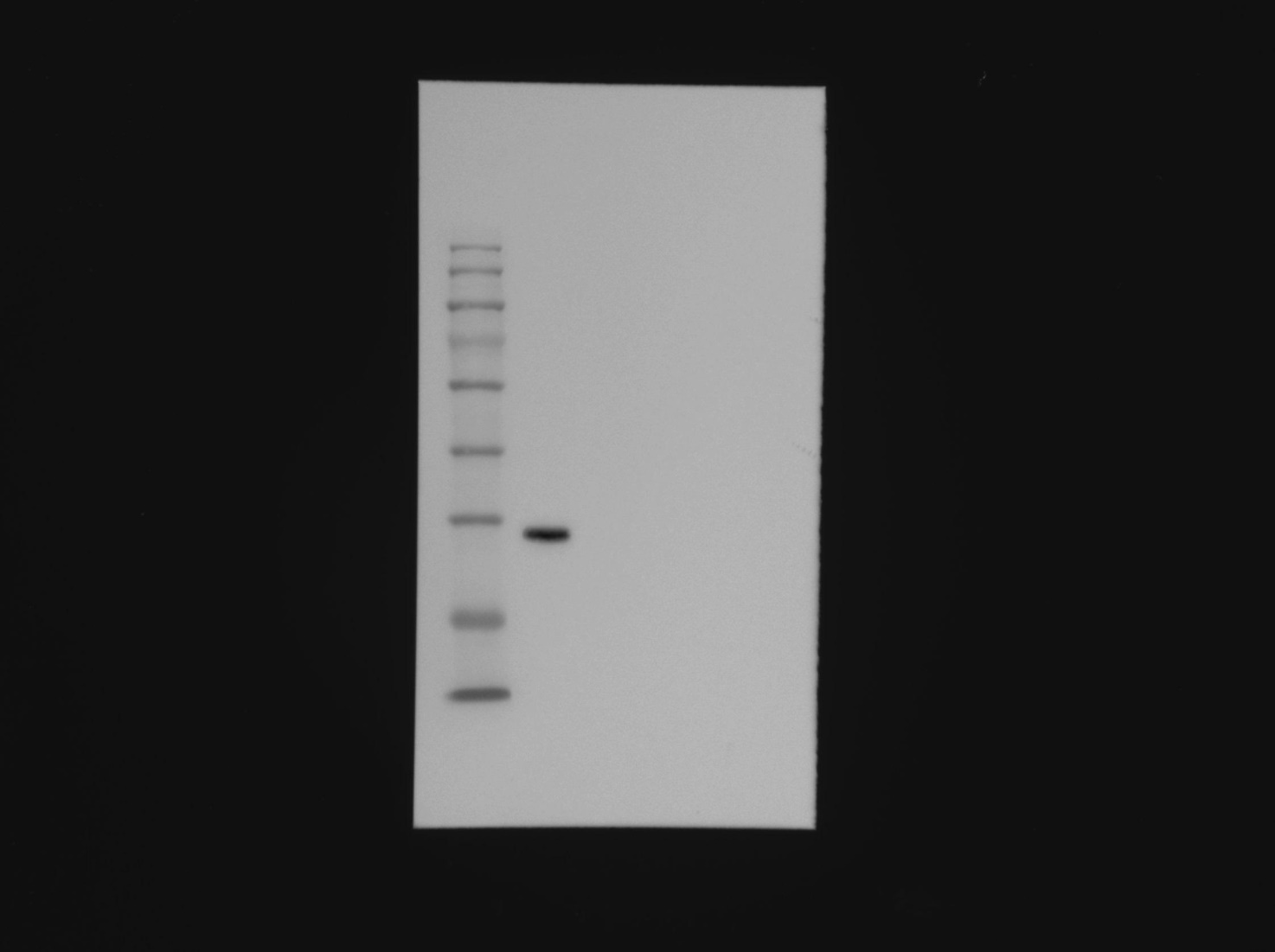


fig4D


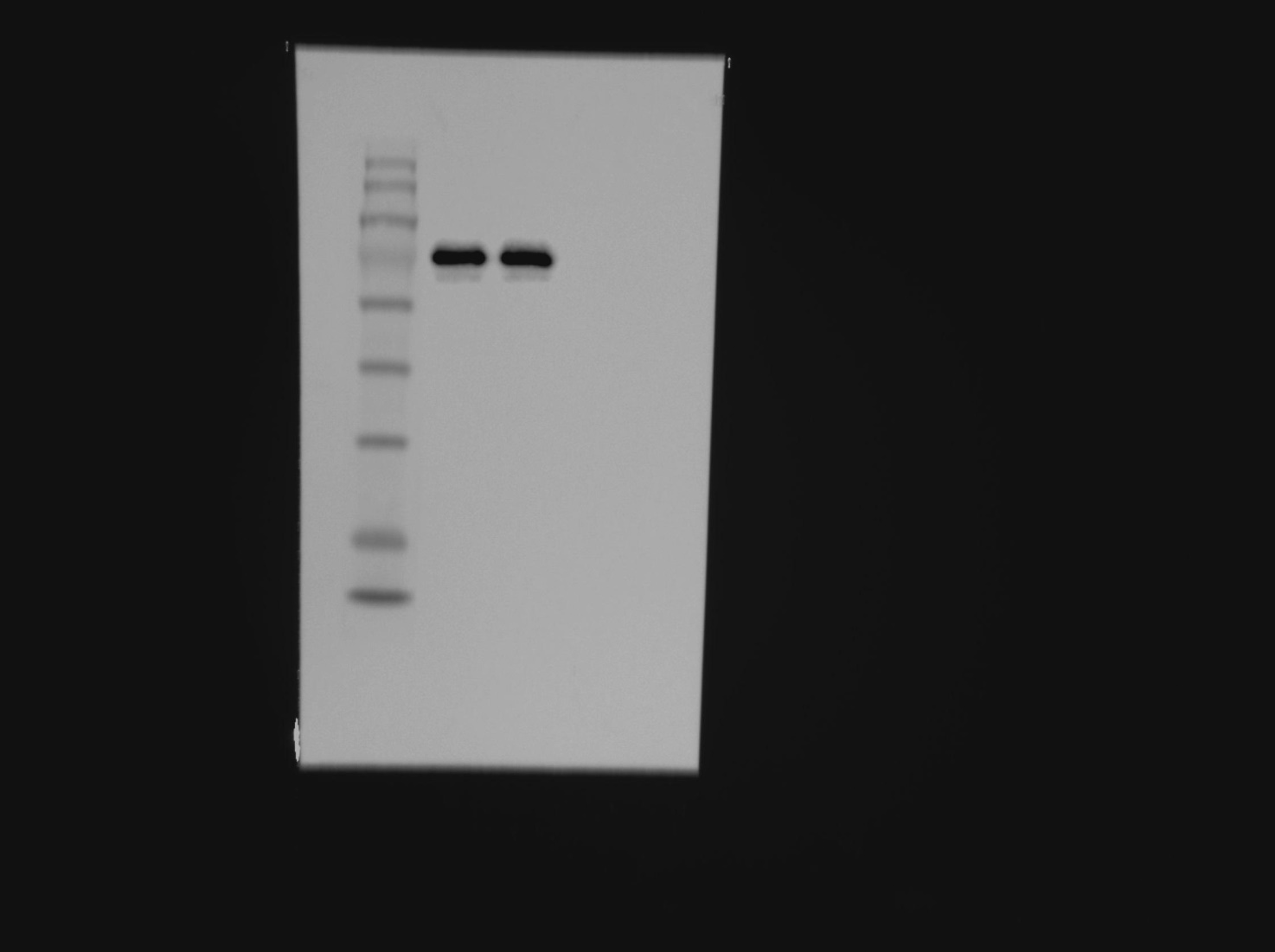


fig4D


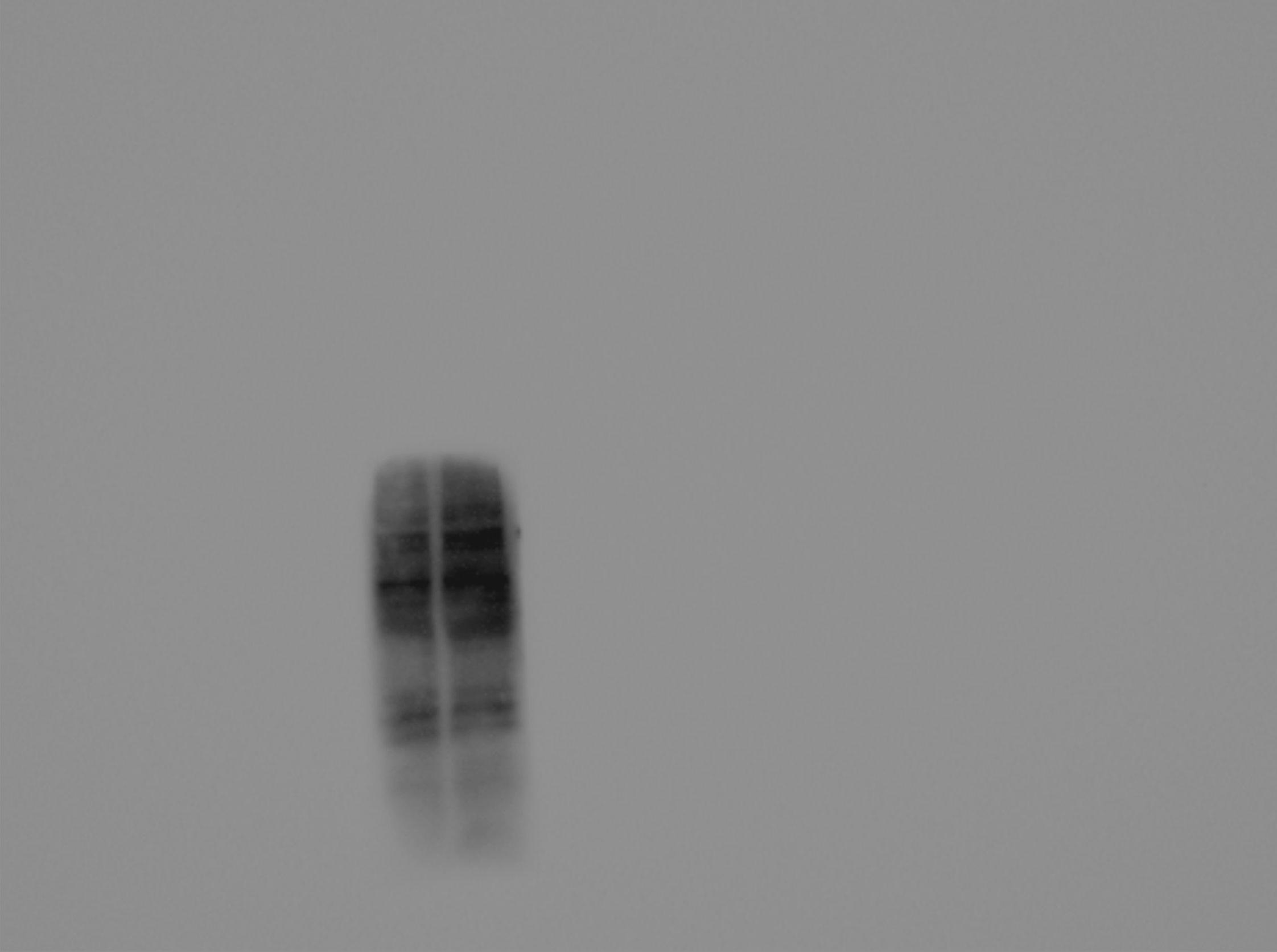


fig4D

Supplement: Supplementary file 1 — Original Data File [file 41420_2022_1119_MOESM1_ESM.docx]
