# Supplementary material for: Deubiquitylase OTUD1 confers Erlotinib sensitivity in non-small cell lung cancer through inhibition of nuclear translocation of YAP1
Source: Cell Death Discov. 2022 Oct 1;8:403. doi: 10.1038/s41420-022-01119-w (PMC9526728; doi:10.1038/s41420-022-01119-w)
Supplement: Supplementary file 2 — Supplementary Table 1 [file 41420_2022_1119_MOESM2_ESM.docx]

**Supplementary Table 1** Primer sequences for reverse transcription quantitative polymerase chain reaction

| Gene | Primer sequence |
| --- | --- |
| OTUD1 | Forward 5’-GACGAGAAGCTGGCCCTATAC-3’ |
|  | Reverse 5’-TGGAATGATGTGGAATCGGTACT-3’ |
| YAP1 | Forward 5’-TAGCCCTGCGTAGCCAGTTA-3’ |
|  | Reverse 5’-TCATGCTTAGTCCACTGTCTGT-3’ |
| SOX9 | Forward 5’-AGCGAACGCACATCAAGAC-3’ |
|  | Reverse 5’-CTGTAGGCGATCTGTTGGGG-3’ |
| GAPDH | Forward 5’-GACTCCACTCACGGCAAATTCA-3’ |
|  | Reverse 5’-TCGCTCCTGGAAGATGGTGAT-3’ |
